# Metabolomics identifies candidate diagnostic biomarkers for pituitary apoplexy

**DOI:** 10.3389/fendo.2026.1879303

**Published:** 2026-09-09

**Authors:** Rui Xu, Shan Xie, Chenxi Li, Bowen Shang, Dongqi Shao, Yaning Chen, Hao Lang, Tianyang Wu, Yu Li, Henian Li, Xialin Zheng, Zhiquan Jiang

**Affiliations:** 1 The First Affiliated Hospital of Bengbu Medical University, Bengbu, Anhui, China; 2 Department of Neurosurgery, the First Affiliated Hospital of Bengbu Medical University, Bengbu, Anhui, China

**Keywords:** biomarkers, glycerol-3-phosphate, N-Acetyl-D-galactosamine, pituitary apoplexy, untargeted metabolomics

## Abstract

**Background:**

Pituitary apoplexy is a clinical emergency resulting from hemorrhage or infarction of a pituitary adenoma, which leads to a sudden increase in tumor volume, acute neuroendocrine dysfunction, and local compressive symptoms. In severe cases, it may be life-threatening. The diagnosis of this condition remains challenging, especially in patients without a prior history of pituitary disease, and reliable serum biomarkers are currently lacking. Metabolomics enables the systematic identification of metabolic alterations under physiological and pathological conditions, thereby providing a powerful tool for elucidating disease mechanisms and discovering diagnostic biomarkers.

**Methods:**

In this study, untargeted metabolomics analysis using UPLC-MS/MS was performed on serum samples collected from 39 patients with nonapoplectic pituitary adenoma and 11 patients with pituitary apoplexy. Principal component analysis (PCA) and orthogonal partial least squares discriminant analysis (OPLS-DA) were applied to assess differences in metabolic profiles between the two groups. Differential metabolites were identified based on variable importance in projection (VIP > 1) and an independent-samples *t*-test (*p* < 0.05). The diagnostic potential of these metabolites was further evaluated using receiver operating characteristic (ROC) curves.

**Results:**

Compared with the nonapoplectic pituitary adenoma group, the pituitary apoplexy group showed distinct serum metabolic profiles, with 92 metabolites upregulated and 310 metabolites downregulated. Further analysis revealed two key differential metabolites—glycerol-3-phosphate (G-3-P) and *N*-acetyl-d-galactosamine (GalNAc)—as potential diagnostic biomarkers for pituitary apoplexy.

**Conclusions:**

This study reveals distinct serum metabolic profiles between the pituitary apoplexy group and the nonapoplectic pituitary adenoma group, offering novel metabolic insights into the pathogenesis of pituitary apoplexy.

## Introduction

Pituitary apoplexy is a clinical emergency resulting from sudden enlargement of a pituitary adenoma due to hemorrhage or infarction, leading to a series of acute neuroendocrine dysfunctions and local compressive symptoms. Sudden-onset severe headache is a typical symptom, which may also be accompanied by nausea, vomiting, and visual field deficits (1–4). As a rare disease, pituitary apoplexy has an incidence of approximately 0.17 cases per 100,000 person-years and an estimated prevalence of approximately 6.2 cases per 100,000 population (5). Magnetic resonance imaging (MRI) offers high diagnostic sensitivity for pituitary apoplexy and can also detect subclinical cases (2, 6). Several hypotheses have been proposed regarding its pathogenesis (7). Previous studies suggest that the occurrence of pituitary apoplexy may be associated with the unique dual blood supply of the pituitary gland. Additionally, a history of major surgery and underlying hemodynamic disorders—such as hypertension, diabetes mellitus, and hyperlipidemia—may promote the onset of apoplexy. Furthermore, molecular factors, including tumor necrosis factor-alpha (TNF-α), vascular endothelial growth factor (VEGF), hypoxia-inducible factor-1 (HIF-1), and matrix metalloproteinases (MMPs), may be involved in its development (8–10). Given that pituitary apoplexy is a potentially life-threatening clinical syndrome, accurate diagnosis and timely intervention are of critical importance.

Metabolomics allows for the qualitative and quantitative profiling of all metabolites in an organism under physiological or pathological conditions using mass spectrometry-based techniques or nuclear magnetic resonance (NMR) spectroscopy, thereby offering insights into the metabolic activities of living systems. This technology has been widely applied to elucidate pathophysiological mechanisms, support disease diagnosis, facilitate drug development, and advance microbial metabolism research (11, 12). Over the past decade, metabolomics has emerged as a core analytical modality for investigations into pituitary neuroendocrine tumors (PitNETs). Compared with conventional single-gene and transcriptomic approaches, metabolomics enables the capture of downstream cellular metabolic reprogramming driven by epigenetic dysregulation and tumor microenvironment remodeling. Integrated multiomics analyses combining genomics, transcriptomics, metabolomics, and gut microbiota sequencing have successfully identified subtype-specific metabolic signatures distinguishing functional and nonfunctional PitNETs. Nevertheless, existing metabolomic studies of pituitary tumors have exclusively focused on stable lesions without acute complications, and systematic reports characterizing circulating metabolic perturbations during the acute phase of pituitary apoplexy remain scarce (13–15).

In the present study, we performed untargeted metabolomics using ultra-performance liquid chromatography-tandem mass spectrometry (UPLC-MS/MS) to characterize serum metabolic profiles in patients with pituitary apoplexy and compare metabolic profiles between apoplectic and nonapoplectic patients, thereby gaining deeper insight into the underlying mechanisms of this disease.

## Methods

### Study design and participants

Patients with pituitary adenoma who underwent surgery in the Department of Neurosurgery, The First Affiliated Hospital of Bengbu Medical University, between August 2024 and August 2025 were enrolled. The cohort included 11 patients with apoplexy and 39 patients without apoplexy. Peripheral blood serum samples were collected upon patient admission. Written informed consent was obtained from all subjects prior to enrollment.

### Sample collection and preparation

Serum samples were thawed in an ice-water bath and transferred into 1.5 mL centrifuge tubes. Protein precipitation was performed by mixing the samples with three volumes of prechilled methanol/acetonitrile (1:1, v/v). After thorough vortexing, the mixture was centrifuged at 4 °C. The resulting supernatant was divided into two aliquots, lyophilized, and stored at − 80 °C until further use.

### Liquid chromatography

Chromatographic separation was performed using a BEH C18 column (1.7 μm, 2.1 mm × 100 mm, Waters, USA). For positive ion mode, the mobile phase consisted of (A) water with 0.1% formic acid and (B) 100% methanol with 0.1% formic acid. For negative ion mode, the mobile phase consisted of (A) water with 10 mM ammonium formate and (B) 95% methanol with 10 mM ammonium formate. Gradient elution was performed as follows: 0–0.1 min, 2% B; 0.1–8 min, 2%–98% B; 8–9.5 min, 98% B; and 9.5–10 min, 2% B. The flow rate was 0.35 mL/min, the column temperature was maintained at 45 °C, and the injection volume was 5 μL.

### Mass spectrometry

MS1 and MS2 data acquisition was performed using an Orbitrap Exploris 480 mass spectrometer (Thermo Fisher Scientific, USA). The mass-to-charge ratio (m/z) scan range was set from 70 to 1,050. The MS1 resolution was 120,000, the AGC target was set to “custom” with a normalized AGC target (%) of 300%, and the maximum injection time (IT) was 100 ms. Based on precursor ion intensity, the top three (Top3) ions were selected for fragmentation to acquire MS2 data. The MS2 resolution was 30,000 (or 30,500 as noted), the AGC target was set to “custom” with a normalized AGC target (%) of 100%, the maximum injection time (IT) was set to “auto”, and the stepped normalized collision energy (stepped NCE) was set to 20, 40, and 60 eV.

The electrospray ionization (ESI) source parameters were set as follows: sheath gas flow rate, 40; auxiliary gas flow rate, 10; spray voltage, 3.80 kV (positive ion mode) and 3.20 kV (negative ion mode); capillary temperature, 320 °C; and auxiliary gas heater temperature, 350 °C.

### Data analysis

Raw UPLC-MS/MS data were processed using TidyMass 2.0 software, which employs a nontargeted metabolomics workflow for peak alignment, feature filtering, gap filling, and metabolite annotation. The parameter settings were as follows: precursor ion mass tolerance, < 5 ppm; fragment ion mass tolerance, < 10 ppm; and retention time tolerance, < 0.2 min. For metabolite identification, the acquired MS/MS spectra were searched against publicly available databases, namely the Human Metabolome Database (HMDB) and Metlin, in conjunction with an in-house library of authentic reference standards. To guarantee data quality and analytical robustness, only metabolic features with detection frequencies exceeding 50% across all samples were retained for downstream statistical analyses.

### Statistical analysis

Data processing was performed using SPSS version 27. Continuous variables were expressed as mean ± standard deviation (*x̄* ± *s*). Comparisons between two groups were conducted using an independent-samples *t*-test, and Cohen’s *d* effect size was calculated. Categorical variables were expressed as counts (percentages), and comparisons between two groups were performed using the Chi-square test or Fisher’s exact test (when the expected cell frequency was < 5). A *p*-value < 0.05 was considered statistically significant.

The MetaboAnalyst online tool was used for data normalization and subsequent analyses, including principal component analysis (PCA), partial least squares discriminant analysis (PLS-DA), and orthogonal partial least squares discriminant analysis (OPLS-DA). Permutation tests were performed to assess the risk of model overfitting in OPLS-DA. Differential metabolites were identified based on variable importance in projection (VIP) scores (> 1.0) from the OPLS-DA model and an independent-samples *t*-test (*p* < 0.05). To control the false discovery rate (FDR) associated with multiple hypothesis testing, the Benjamini–Hochberg (BH) procedure was applied to adjust the *p*-values of all detected metabolic features. An adjusted *p*-value (*q*-value) < 0.05 was considered statistically significant for metabolite selection, in addition to the VIP > 1 criterion. The total number of features detected in the serum metabolomic profiling was also recorded to provide context for the multiple comparison correction. Receiver operating characteristic (ROC) curve analysis was performed to evaluate the diagnostic potential of key metabolic alterations, and Spearman’s correlation coefficient was calculated for correlation analysis. To rigorously assess the discriminative performance and mitigate overfitting bias, we performed leave-one-out cross-validation (LOOCV) at two levels. First, to evaluate the generalizability of the multivariate OPLS-DA model, LOOCV was conducted on the full metabolic model: in each iteration, one sample was excluded as the validation set, and the remaining 49 samples were used to construct the OPLS-DA model, with the predicted probability for the excluded sample being recorded. This process was repeated until every sample had served once as the validation set. The resulting 50 cross-validated predicted probabilities were used to generate a bias-corrected ROC curve, providing a more conservative estimate of the overall discriminative performance of the model. Second, for the two individual candidate biomarkers (glycerol-3-phosphate [G-3-P] and *N*-acetyl-d-galactosamine [GalNAc]), separate LOOCV was performed using univariate ROC models, and bias-corrected area under the curve (AUC) values were derived for each metabolite.

## Results

### Baseline demographic and clinical data

This study included 11 patients with pituitary apoplexy and 39 patients with nonapoplectic pituitary adenoma. The two groups were well matched with respect to gender, age, tumor diameter, and the prevalence of hypertension and diabetes mellitus (all *p* > 0.05). Regarding pathological types, the proportion of functional adenomas did not differ significantly between the apoplexy and nonapoplexy groups (two of 11, 18.2% vs. 8/39, 20.5%; Fisher’s exact test, *p* = 1.000). Among functional subtypes, the apoplexy group comprised two prolactin-secreting adenomas and nine nonfunctional adenomas, while the nonapoplexy group included five prolactin-secreting, three growth hormone (GH)-secreting, and 31 nonfunctional adenomas; the overall subtype distribution also showed no significant difference between groups (Fisher’s exact test, *p* = 0.481).

Hormone-related symptoms included galactorrhea/amenorrhea in patients with prolactin (PRL)-secreting adenomas and acromegaly in GH-secreting cases (**Table 1**).

**Table 1:** Demographic data of patients with pituitary apoplexy and nonapoplectic pituitary adenoma.

Variable	Apoplexy group (*n* = 11)	Nonapoplexy group (*n* = 39)	Statistical value (*χ*^2^/t)	*P*-value
Gender (man/woman)	5/6	17/22	–	1.00
Age (years, mean ± standard deviation)	49.36 ± 14.00	51.15 ± 13.55	− 0.38	0.71
Hypertension (yes/no)	4/7	11/28	–	0.71
Diabetes (yes/no)	1/10	3/36	–	1.00
Tumor diameter (maximum diameter) (mm, mean ± standard deviation)	28.70 ± 10.19	25.66 ± 12.42	0.74	0.46
Pathological type				
Functional	2	8		1.00
Nonfunctional	9	31		
Functional subtype				
Prolactin-secreting	2	5		0.481
GH-secreting	0	3		
Nonfunctional	9	31		
Hormone-related symptoms				
Galactorrhea/Amenorrhea (PRL)	1/2	2/5		
Acromegaly (GH)	0	1/3		
Acute onset symptoms				
Sudden severe headache (yes/no)	10/1	5/34		
Visual field defect (yes/no)	7/4	6/33		

### Statistical analysis of serum metabolic profiles in patients with pituitary apoplexy and nonapoplectic controls

**Figure 1:**
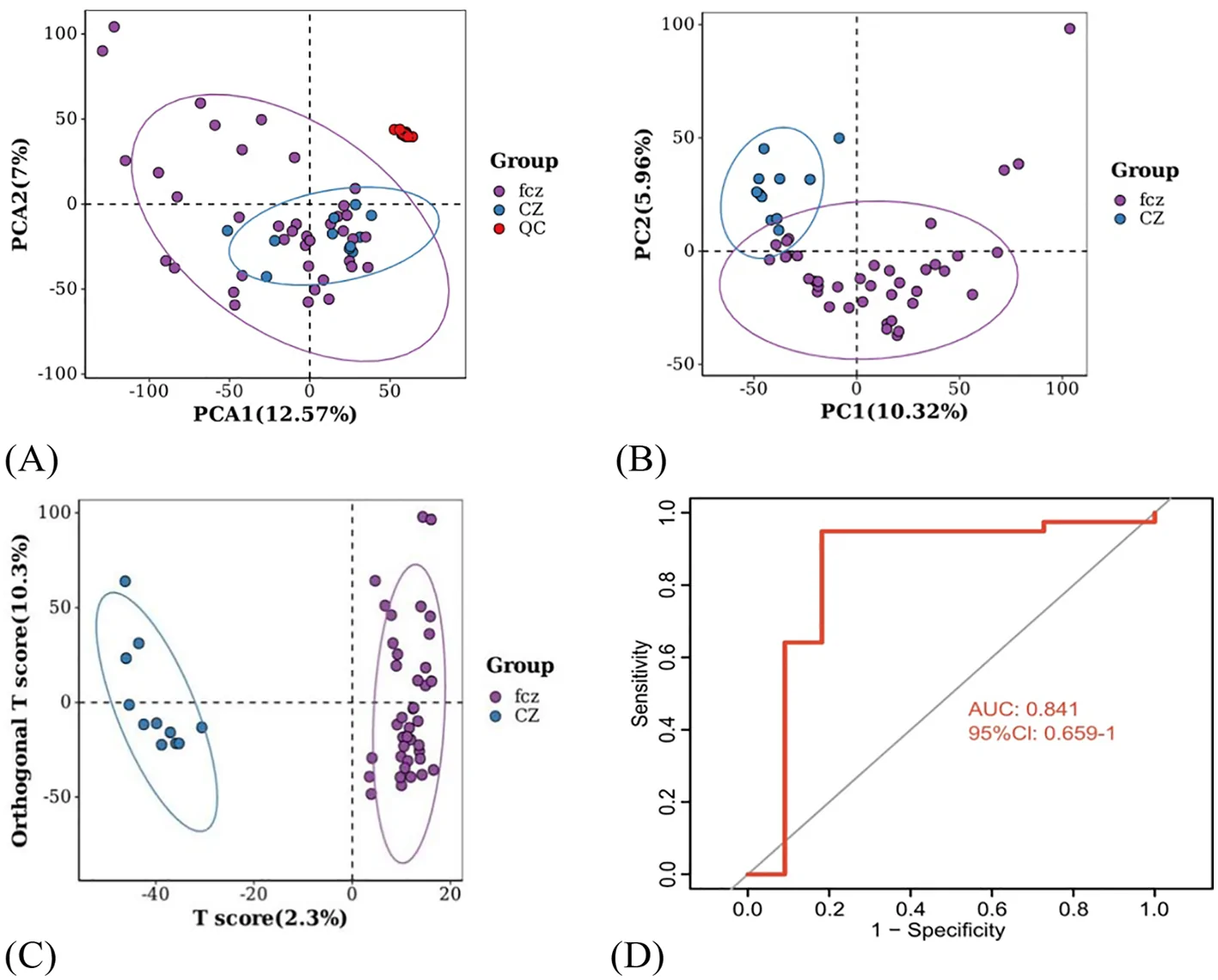
Statistical analysis of the serum metabolome: **(A)** PCA score plot. Quality control (QC) samples are tightly clustered, indicating minimal systematic error and high experimental data quality; **(B)** PLS-DA score plot. There is a clear trend toward differentiation in serum metabolite profiles between the pituitary tumor apoplexy group and the nonapoplexy group; **(C)** OPLS-DA score plot, showing significant intergroup metabolomic differences and moderate intra-group variation. FCZ, pituitary tumor nonapoplexy group; CZ, pituitary tumor apoplexy group; **(D)** bias-corrected receiver operating characteristic (ROC) curve of the OPLS-DA model for distinguishing pituitary apoplexy from nonapoplexy. The area under the curve (AUC) was 0.841 (95% CI: 0.659–1.000), indicating moderate discriminative ability after internal validation.

Serum samples from each group were subjected to UPLC-Q-Exactive-Orbitrap-MS analysis to generate metabolic profiles. PCA was used to assess the overall distribution of metabolic profiles and experimental reproducibility. As shown in **Figure 1A**, the quality control (QC) samples were tightly clustered around the origin of the PCA score plot, reflecting favorable system stability and low operational variability.

As shown in **Figure 1B**, the apoplexy and nonapoplexy groups exhibited a clear separation trend, indicating differences in metabolite profiles between the two cohorts. To further explore these metabolic differences while minimizing overfitting risk, we performed OPLS-DA (**Figure 1C**). The OPLS-DA score plot showed complete separation between the nonapoplectic pituitary adenoma group (purple dots) and the pituitary apoplexy group (blue dots) on the plane defined by the predictive component (*T* score, 2.3%) and the orthogonal component (orthogonal *T* score, 10.3%), with no overlapping samples. The tight within-group clustering indicates robust and significant metabolic differences between the two groups.

To further assess the generalizability of the OPLS-DA model and correct for potential optimistic bias in its discriminative performance, we performed LOOCV as an internal validation. The 50 cross-validated predicted probabilities were then used to generate a bias-corrected ROC curve, yielding a corrected AUC of 0.841 (95% CI: 0.659–1.000), suggesting that the metabolic model retains moderate discriminative ability after internal validation (**Figure 1D**). However, the diagnostic threshold and clinical generalizability of the model require further validation in independent external cohorts. These findings indicate that the serum metabolic profiles differ reliably between the apoplexy and nonapoplexy groups, providing a reproducible basis for subsequent differential metabolite screening.

### Serum metabolic alterations: pituitary apoplexy vs. nonapoplectic patient

**Figure 2:**
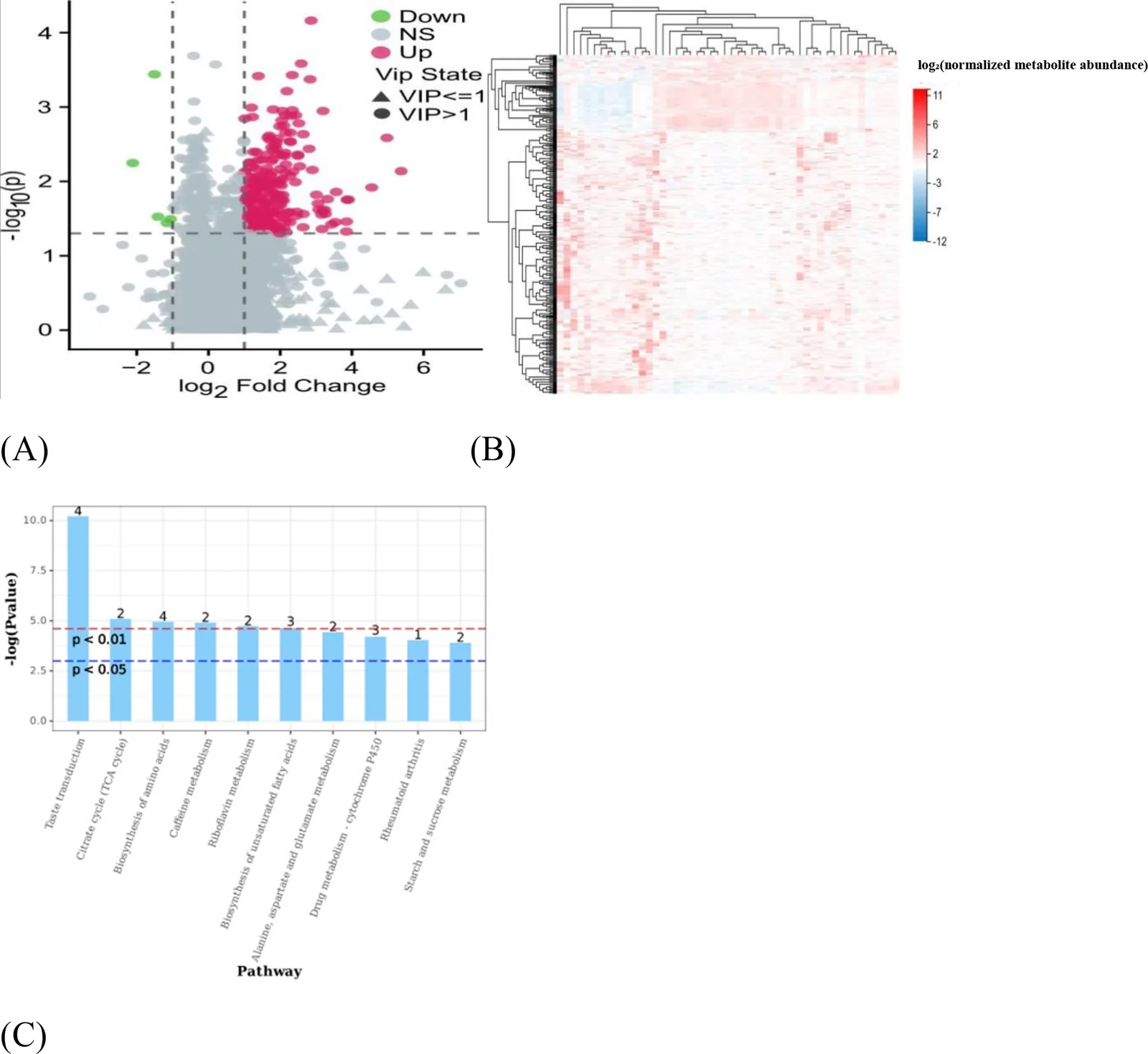
Serum metabolomics analysis of patients with pituitary tumor apoplexy and those without apoplexy: **(A)** volcano plot of differentially expressed serum metabolites after false discovery rate (FDR) correction. Green represents significantly downregulated metabolites in the nonapoplexy group compared with the apoplexy group, red represents significantly upregulated metabolites in the nonapoplexy group compared with the apoplexy group, and gray represents nonsignificant metabolites. Circles indicate metabolites with VIP ≥ 1, and triangles indicate metabolites with VIP < 1. **(B)** Heatmap of differential metabolites (each row represents a differential metabolite, and each column represents a sample; the color bar on the right shows log_2_-transformed normalized metabolite abundance (red, high abundance; blue, low abundance, range: − 12 to 11). **(C)** Metabolic pathway enrichment analysis (the *y*-axis represents –log_10_(*p*-value), with higher values indicating stronger enrichment significance. Numbers at the top of each bar denote the number of significantly differentially enriched metabolites in the corresponding KEGG pathway. Metabolic pathways with *p* < 0.05 were defined as significantly enriched pathways for differential metabolites.

VIP values were calculated from the OPLS-DA model to quantify the contribution of each variable to group discrimination. A total of 4,551 metabolic features were detected in the serum metabolomic profiling. Before FDR correction, 402 metabolites showed significant intergroup differences (VIP > 1, unadjusted *p* < 0.05), including 92 upregulated and 310 downregulated metabolites. After Benjamini–Hochberg correction for multiple comparisons (*q* < 0.05), 240 metabolites remained statistically significant. Among these, *N*-acetyl-d-galactosamine and glycerol-3-phosphate were consistently identified as differentially expressed metabolites under both criteria, supporting their selection as candidate biomarkers (**Figures 2A, B**).

To explore altered metabolic pathways in patients with pituitary apoplexy compared with those without, we performed pathway enrichment analysis on the candidate metabolites using MetaboAnalyst 6.0, aiming to identify relevant metabolic pathways and potential biological functions. The results revealed that these differentially expressed metabolites were primarily enriched in pathways such as taste transduction, the tricarboxylic acid (TCA) cycle, amino acid biosynthesis, and starch and sucrose metabolism (**Figure 2C**).

### Key metabolites identified for distinguishing pituitary apoplexy from nonapoplectic pituitary adenoma

**Figure 3:**
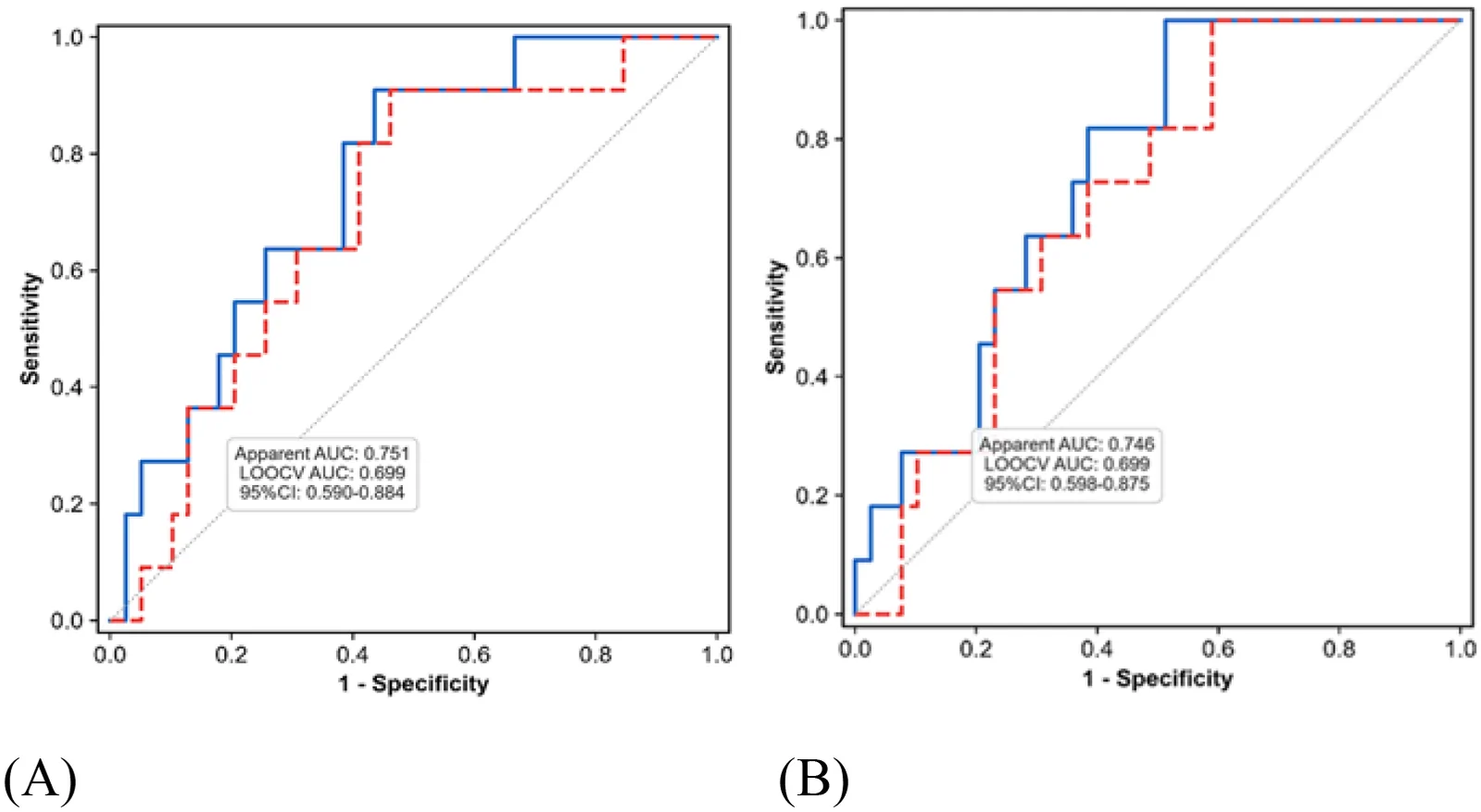
Receiver operating characteristic (ROC) curves of the two key differential metabolites with leave-one-out cross-validation (LOOCV) correction. **(A)** *N*-acetyl-d-galactosamine; **(B)** glycerol-3-phosphate. The blue solid lines represent the initial ROC curves, whereas the red dashed lines represent the bias-corrected ROC curves derived from LOOCV.

Among the differential metabolites identified between the pituitary apoplexy and nonapoplexy groups, two key metabolites—*N*-acetyl-d-galactosamine and glycerol-3-phosphate—were selected for further evaluation. ROC curve analysis was used to assess their diagnostic potential, yielding initial AUC values of 0.751 and 0.746, respectively. To mitigate potential overfitting bias, we applied LOOCV to correct the performance estimates. The bias-corrected AUC values were 0.699 and 0.699, respectively, indicating that both metabolites retained moderate discriminative ability after internal validation and may serve as potential adjunctive biomarkers (**Figure 3**).

To explore serum metabolic differences between patients with pituitary apoplexy and those with nonapoplectic pituitary adenoma, we examined the key metabolites that were significantly altered between the two groups. The levels of *N*-acetyl-d-galactosamine and glycerol-3-phosphate were significantly lower in the pituitary apoplexy group, suggesting their potential role in distinguishing patients with apoplexy from those without apoplexy.

## Discussion

Pituitary apoplexy is a rare but potentially life-threatening clinical syndrome if not promptly treated. Its underlying mechanisms remain poorly understood, and diagnosis remains challenging, especially in patients without a prior history of pituitary disease. While neuroimaging is essential for evaluating pituitary lesions, current modalities have notable limitations. Computed tomography (CT) can diagnose acute-stage pituitary apoplexy, yet hemorrhage is detectable in only 21%–28% of patients. MRI confirms the diagnosis in 88% of cases but fails to identify acute hemorrhage during the acute phase (7).

Currently, no reliable biomarkers are available for the diagnosis of pituitary apoplexy. Serum, which reflects systemic metabolic changes, is widely used in clinical metabolomics. In this study, preoperative serum-based metabolomic analysis revealed significant metabolic differences between patients with pituitary apoplexy and those with nonapoplectic pituitary adenoma, leading to the identification of two differential metabolites.

G-3-P is a key backbone molecule for the biosynthesis of glycerophospholipids and triglycerides. Neuronal cell membranes are rich in glycerophospholipids. During acute neurological injury, such as cerebral ischemia and hypoxia, excessive activation of phospholipases leads to extensive degradation of membrane phospholipids, disrupting membrane integrity. Under these conditions, G-3-P—an important substrate for phospholipid resynthesis—decreases due to increased consumption and impaired synthesis. Beyond their structural role in membranes, glycerophospholipids and their metabolites are actively involved in inflammatory and immune modulation. In particular, ether-type glycerophospholipids exert anti-inflammatory and neuroprotective effects by inhibiting microglial activation and the expression of inflammatory cytokines. 1-*O*-Alkylglycerophosphate (AGP), a key intermediate in the synthesis of ether-type glycerophospholipids, serves as a partial agonist of peroxisome proliferator-activated receptor gamma (PPARγ). PPARγ is a ligand-inducible nuclear receptor that promotes the anti-inflammatory M2 phenotype in macrophages and microglia. Previous studies have shown that PPARγ activation induces apoptosis in various pituitary tumor cell lines and reduces the levels of adrenocorticotropic hormone (ACTH), PRL, and GH. Therefore, we hypothesize that the decrease in G-3-P levels following pituitary apoplexy may reduce AGP synthesis, thereby attenuating PPARγ pathway activation. This could, on one hand, diminish PPARγ-mediated anti-inflammatory and neuroprotective effects and, on the other hand, inhibit PPARγ-induced apoptosis of pituitary tumor cells. Such dual effects may contribute to exacerbated postapoplexy inflammation and influence tumor prognosis (16). This hypothesis warrants further validation through intervention experiments using PPARγ agonists or inhibitors.

GalNAc is a basic unit of complex sugar chains in the body. It is widely present in glycoproteins and glycolipids on the cell membrane surface, playing critical roles in protein stability, cell recognition, and signal transduction. Complex gangliosides promote the secretion of extracellular vesicles (EVs) via GalNAc residues at the terminal ends of their sugar chains, a process essential for neurons to clear misfolded proteins and maintain protein homeostasis (17). Previous studies have shown that in pituitary adenoma—a benign tumor—EVs can reduce cell motility or alter the tumor microenvironment to attenuate metastasis (18). In this study, serum GalNAc levels were significantly decreased in patients with pituitary apoplexy. We hypothesize that this reduction may weaken EV-mediated neuroprotective mechanisms by decreasing the synthesis of complex gangliosides, thereby contributing to the pathological process of neurological injury following apoplexy. Given that EVs are not only involved in protein clearance but also play roles in inflammatory regulation and intercellular communication, the pathological significance of reduced GalNAc levels warrants further in-depth investigation in pituitary apoplexy models.

In summary, this study used UPLC-MS/MS metabolomics to reveal significantly altered serum levels of G-3-P and GalNAc in patients with pituitary apoplexy. These two metabolites offer new insights into the pathogenesis of pituitary apoplexy in two key areas: membrane phospholipid metabolism and anti-inflammatory signaling, and glycoconjugate-mediated protein homeostasis. These metabolites may serve as potential adjunctive diagnostic biomarkers, and future studies should further evaluate the diagnostic value of their combined detection.

Nevertheless, several limitations of this study should be acknowledged. First, the sample size is relatively small, and this study did not construct a formal diagnostic prediction model based on the screened differential metabolites. We only identified two potential metabolic biomarkers (G-3-P and GalNAc) and performed LOOCV to preliminarily evaluate the diagnostic potential of the metabolic signatures and correct for optimistic bias in the performance estimates. Notably, such internal validation cannot replace external validation in independent cohorts, and the lack of a systematic prediction model also limits the quantitative evaluation of the diagnostic efficacy and clinical generalization ability of the two metabolites. Therefore, the clinical diagnostic value of these metabolic biomarkers needs to be further validated by constructing a rigorous prediction model in larger, prospective, multi-center studies. Second, the absence of metabolomic data from tumor tissues and the local microenvironment limits our ability to fully interpret the origins and biological relevance of the differentially expressed metabolites. Future studies integrating animal models and cellular experiments are needed to further elucidate the causal relationships and underlying molecular mechanisms of these metabolic alterations.

## Conclusions

This study expands our understanding of serum metabolomic alterations in pituitary apoplexy. Several metabolites correlated with key clinical parameters, highlighting the potential of serum metabolomics for diagnostic modeling. These findings provide new insights into the diagnosis and identification of potential therapeutic targets for pituitary apoplexy.

## Data Availability

The raw data supporting the conclusions of this article will be made available by the authors, without undue reservation.
